# Interleukin-17 Contributes to the Pathogenesis of Autoimmune Hepatitis through Inducing Hepatic Interleukin-6 Expression

**DOI:** 10.1371/journal.pone.0018909

**Published:** 2011-04-19

**Authors:** Li Zhao, Yanli Tang, Zhengrui You, Qixia Wang, Shuwen Liang, Xiaofeng Han, Dekai Qiu, Jue Wei, Yuan Liu, Lei Shen, Xiaoyu Chen, Yanshen Peng, Zhiping Li, Xiong Ma

**Affiliations:** 1 Department of Gastroenterology, Renji Hospital, Shanghai Jiao Tong University School of Medicine, Shanghai Institute of Digestive Disease, Shanghai, China; 2 Department of Medicine, Johns Hopkins University, Baltimore, Maryland, United States of America; University of Leuven, Rega Institute, Belgium

## Abstract

T helper cells that produce IL-17 (Th17 cells) have recently been identified as the third distinct subset of effector T cells. Emerging data suggests that Th17 cells play an important role in the pathogenesis of many liver diseases by regulating innate immunity, adaptive immunity, and autoimmunity. In this study, we examine the role and mechanism of Th17 cells in the pathogenesis of autoimmune hepatitis (AIH). The serum levels of IL-17 and IL-23, as well as the frequency of IL-17+ cells in the liver, were significantly elevated in patients with AIH, compared to other chronic hepatitis and healthy controls. The hepatic expressions of IL-17, IL-23, ROR-γt, IL-6 and IL-1β in patients with AIH were also significantly increased and were associated with increased inflammation and fibrosis. IL-17 induces IL-6 expression via the MAPK signaling pathway in hepatocytes, which, in turn, may further stimulate Th17 cells and forms a positive feedback loop. In conclusion, Th17 cells are key effector T cells that regulate the pathogenesis of AIH, via induction of MAPK dependent hepatic IL-6 expression. Blocking the signaling pathway and interrupting the positive feedback loop are potential therapeutic targets for autoimmune hepatitis.

## Introduction

Autoimmune hepatitis (AIH) is an inflammatory liver condition characterized by interface hepatitis, hypergammaglobulinemia, serum autoantibodies, and satisfactory response to immunosuppressive treatment [1], [2]. Immune reactions against host liver antigens are believed to be a major pathogenic mechanism. The histological feature of interface hepatitis, with the infiltration of lymphocytes, plasma cells, and macrophages, suggests an aggressive cellular immune response in the pathogenesis of AIH [3]. Immunohistochemical studies have identified the predominant T lymphocyte infiltration as CD4^+^ helper/inducer T cells and CD8+ T cells [4]. Following their activation, naïve CD4^+^ T cells differentiate into distinct T helper cell lineages, including pro-inflammatory Th1 cells, anti-inflammatory Th2 cells, regulatory T cells (Tregs) and newly defined Th17 cells [5]. Autoimmune hepatitis has been associated with predominating Th1 responses and decreased frequency and function of Tregs [6], [7].

The recently identified Th17 cells are CD4+ T cells characterized by the secretion of IL-17 [8]. The cytokine transforming growth factor–β (TGF-β), in the presence of interleukin-6 (IL-6), promotes the differentiation of naïve T lymphocytes into Th17 cells, which promote autoimmunity and inflammation [9]. Since liver is known to be an important source of TGF-β and IL-6, Th17 differentiation may be favored in the liver. In fact, Th17 cells have been found to be involved in primary biliary cirrhosis [10] and alcoholic liver disease [11], injuries known to result from increased levels of TGF-β and IL-6. In contrast, little is known about the role of Th17 cells on the pathogenesis of AIH, though, deletion of Th17 has been shown to reduce T cell mediated hepatitis [12]. The aim of the current study is to investigate whether Th17 cells and/or the IL-17 signaling pathway play a role in the pathogenesis of AIH. Additionally, this study examines the potential mechanisms that underline Th17 mediated liver inflammation in AIH. Results from this study may have profound therapeutic implications for the management of autoimmune liver disease.

## Materials and Methods

### Patients and samples

Patients who satisfied the international pretreatment criteria for the definitive diagnosis of autoimmune hepatitis (AIH) type I were included in the study, in addition to patients with chronic hepatitis B (CHB) and healthy volunteers. All participants gave written informed consent for the study that was approved by the Ethics Committee of Renji Hospital, Shanghai Jiao Tong University. Peripheral blood samples from 29 AIH patients, 18 CHB patients and 28 healthy controls; liver biopsy samples from 39 patients, 32 CHB patients and 5 healthy controls and fresh frozen liver tissue from 28 AIH patients, 12 CHB patients and 5 healthy were included in the study. The characterization of participants is shown in Table 1. Peripheral blood mononuclear cells (PBMCs) were isolated using a Ficoll separation (Ficoll-Paque plus; Amersham Bioscience, Shanghai, China). PBMCs were then labeled with direct fluorescent conjugated anti-human antibodies against CD3, CD4, CD25, CCR4 and CCR6 (BD Pharmingen, San Diego, CA). PBMCs were evaluated by flow cytometry (Calibur, Becton Dickinson), and the data were analyzed using Cell Quest software (Becton Dickinson). Serum IL-17A and IL-23 (p19/p40) were measured by ELISA (eBioscience, San Diego, USA). Total RNA was extracted from biopsy samples using Trizol reagent (Invitrogen, Carlsbad, CA, USA). Complementary DNA was synthesized from 0.5 µg of total RNA using oligo(dT) as the template and the RevertAid™ First Strand cDNA Synthesis Kit (Fermentas, Canada). Real-time PCRs were performed using SyBR Premix Ex Taq TM (Takara) in a Roche LightCycler 480. Negative controls were performed without cDNA in the reaction mixture. The results were normalized against glyceraldehyde-3-phosphate dehydrogenase (GAPDH) gene expression. The primer sequences for IL-17, IL-23 (p19), IL-21, IL1-B, IL-6 and GAPDH are described in Table 2. Liver biospies were fixed in 10% neutral buffered formalin, embedded in paraffin, and 4 µm thick sections were cut from each paraffin block. The slides were stained with hematoxylin-eosin, and inflammatory degree and fibrotic stages were determined according to the Scheuer score system. For immunohistochemistry, the sections were pre-incubated with 5% bovine serum albumin for 10 min, and then incubated with anti-human IL-17 antibody (1:50 dilution, R&D systems, Minneapolis, MN, USA) for 12 hours at 4°C in a wet chamber. After washing with phosphate buffer saline (PBS), the sections were incubated with an anti-goat secondary antibody (Zhongshan Golden Bridge Biotech, Beijing, China) for 20 min at room temperature, and detected with diaminobenzidine (DAB) and hematoxylin as the counter stain. For immunofluorescent staining, liver specimens were incubated with antibodies for CD4 (Shanghai Changdao Biotech, Shanghai, China) and IL-17 (R&D), and imaged with confocal microscopy (Leica TCS SP5II, Wetzlar, Germany).

**Table 1:** Characterization of the Study Participants.

	Healthy Control (HC, n = 28)	Chronic Hepatitis B (CHB, n = 32)	Autoimmune Hepatitis (AIH, n = 39)
Sex (female/male)	22/6	25/7	32/7
Age (years)	49 (22–58)	45 (18–60)	51 (18–64)
ALT	19 (9–31)	68 (18–242)	72 (24–238)
AST	27 (11–45)	62 (21–248)	68 (26–225)
Histological Findings			
Inflammation (1/2/3/4)	NA	7/14/10/1	2/20/15/2
Fibrosis (0/1/2/3/4)	NA	9/6/4/5/8	5/8/6/14/6

Data is shown as media and range. ALT: alanine aminotransferase; AST: aspartate aminotransferase. There is no statistical difference between all three groups in sex, age. There is no statistical difference between CHB and AIH groups in serum transaminases and histological findings.

**Table 2:** The primers sequences of Th17 cells related genes and GAPDH.

Gene	Forward primer	Reverse primer
IL-17	TGTCCACCATGTGGCCTAAGAG	GTCCGAAATGAGGCTGTCTTTGA
IL-23 (p19)	CCAAGGACTCAGGGACAACA	ATCAGGGAGCAGAGAAGGCT
IL-21	GCCACATGATTAGAATGCGTCAAC	TGGAGCTGGCAGAAATTCAGG
IL-1β	GCTGATGGCCCTAAACAGATGAA	TGAAGCCCTTGCTGTAGTGGTG
RORγt	CATCTCCAGCCTCAGCTTTGA	CATCTCCAGCCTCAGCTTTGA
IL-6	AAGCCAGAGCTGTGCAGATGAGTA	TGTCCTGCAGCCACTGGTTC
GAPDH	GAAGGTGAAGGTCGGAGTC	GAAGATGGTGATGGGATTTC

For each sample, mRNA expression level was normalized to the level of GAPDH housekeeping genes using the ΔΔCt algorithm.

### Cell culture and ELISA assay

Human hepatoma HepG2 cells were cultured in DMEM with 10% fetal bovine serum (complete medium). At 70% confluence, the complete medium was replaced with media containing 0.5% FBS. After overnight incubation (quiescent cells), IL-17 (with a final concentration of 1, 10, and 100 ng/ml) was added and the cells were cultured for an additional 12, 24 or 48 hours. At the end of the experiment, culture media were collected and IL-6 and monocyte chemotactic peptide-1 (MCP-1) in the media was determined with a standard ELISA according to the manufacturer's instructions (R&D Systems, Minneapolis, MN, USA). For kinase inhibition experiments, HepG2 cells were pretreated with specific inhibitors to p38 MAPK (SB203580), p42/p44 ERK (PD98059 and U0126) and JNK (SP600125) (Calbiochem, Schwalbach, Germany), for 30 min before IL-17 (100 ng/ml) was added to the media. The cells were cultured for an additional 48 hours.

### Western Blotting

Total protein extractions were made from HepG2 cells using a protein extraction kit (Beyotime Institute of Biotechnology). The protein concentration was determined by the BCA assay (Beyotime Institute of Biotechnology). Aliquots containing 30 µg of protein were subjected to electrophoresis in 10% SDS-PAGE, followed by transfer to nitrocellulose membranes. The membranes were blocked with 5% nonfat dry milk in Tris- buffered saline (TBST), 0.1% Tween 20 for 1 h at room temperature, and then incubated overnight at 4°C with antibodies specific against phosphorylated ERK1/2, p38 MAPK, JNK (Cell Signaling Technology, Beverly, MA, USA). After extensive washing, the blots were incubated with a secondary antibody and signals were detected with an ECL detection kit (Pierce, Rockford, IL, USA). The membranes were then stripped and re-probed with an antibody against total ERK, p38 MAPK and JNK. The ratio of the expression levels of the phosphorylated ERK1/2, p38 MAPK, JNK to those of total ERK, p38 MAPK, JNK were taken to represent their activity, respectively. β-actin expression was determined as loading controls.

### Statistical analysis

All values are expressed as mean ± SD. Correlations were determined by Spearman's correlation coefficient (SPSS13.0). The group means were compared by t-test using Microsoft Excel (Microsoft, Redmond, WA). *P* values of less than 0.05 were considered statistically significant.

## Results

### Increased IL-17 and IL-23 levels and Th17 cell frequency in the peripheral blood of patients with AIH

**Figure 1:**
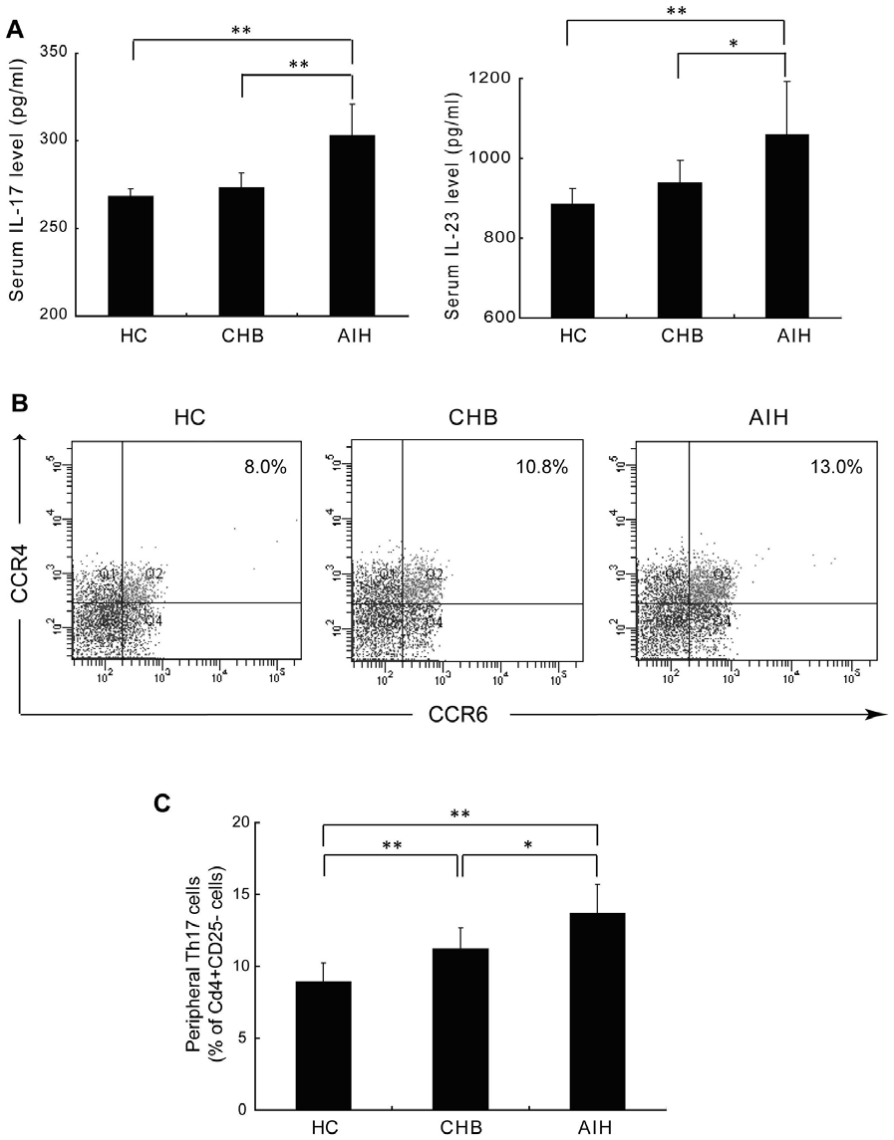
Blood samples were obtained from healthy controls (HC, n = 28), chronic hepatitis B (CHB, n = 18) and autoimmune hepatitis (AIH, n = 29) patients. Peripheral blood mononuclear cells (PBMCs) were isolated, labeled with fluorescent antibodies against CD4, CD25, CCR4 and CCR6, and analyzed by flow cytometry. (A) Plasma IL-17 and IL-23 levels. (B) Representative dot plots; and (C) Mean (±SD) percentage of Th17 (CD4^+^CD25^−^ CCR4^+^CCR6^+^) cells in PBMC. Panel B and C are gated on CD4^+^CD25^−^ cells. *p<0.05, **p<0.01.

Th17 cells are characterized by producing large quantities of IL-17A (also called IL-17) that is attributed to most Th17 cell-mediated effects. IL-23 plays a critical role in the expansion and stabilization of Th17 cells *in vitro* and *in vivo*. To determine whether Th17 cells play a role in the pathogenesis of AIH, we first evaluated serum IL-17 and IL-23 (p19/p40) levels, and the relative frequency of Th17 cells in the peripheral blood of patients with AIH, CHB and healthy controls. We found that plasma IL-17 and IL-23 levels were significantly increased in patients with AIH, compare to those in healthy controls or patients with CHB (Figure 1A). There was no difference in plasma IL-17 and IL-23 levels between patients with CHB and healthy controls.

The percentage of Th17 cells in peripheral CD4^+^ T cell populations was determined by flow cytometry. We defined Th17 cells as CCR4^+^CCR6^+^CD4^+^CD25^−^ T cells according to previous study [13]. There was a significant increase in Th17 cells among PBMCs from AIH patients, compared to those from healthy control or CHB patients (Figure 1B, C). These results indicate that there is an expanded Th17 cell population and an increased IL-17 level in the peripheral blood of AIH patients compared to normal individuals, as well as patients with other chronic hepatitis.

### Increased Th17 cells and related cytokines in the liver of AIH patients

**Figure 2:**
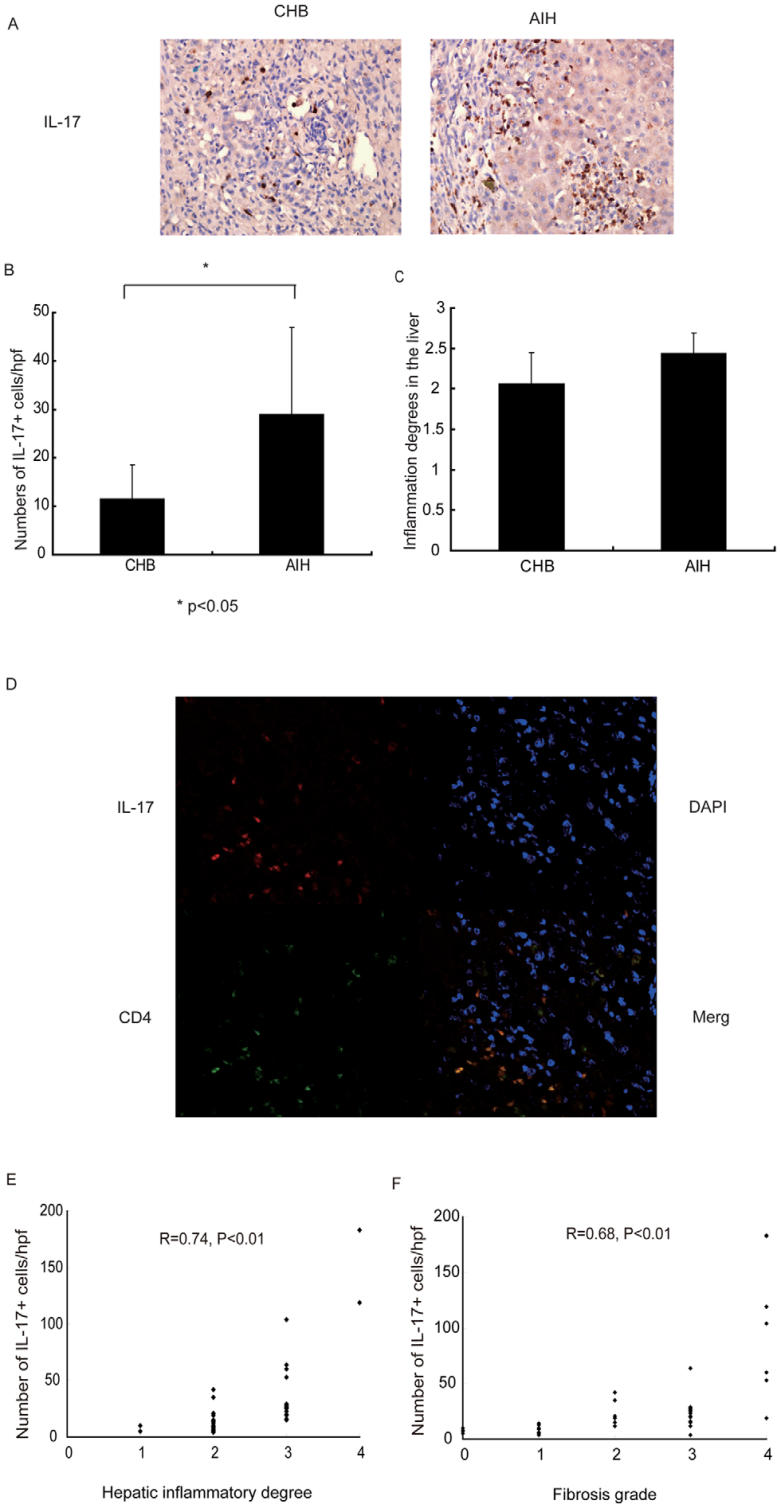
Liver biopsies were obtained from patients with either autoimmune hepatitis (AIH, n = 39) or chronic hepatitis B (CHB, n = 32). Th17 cells in the liver were evaluated by immunohistochemical staining of IL-17. (A) Representative histology of Th17 cells (IL-17+, brown stained cells, 400×); (B) Mean (±SD) of Th17 cells in AIH and CHB patients; (C) Mean (±SD) of hepatic inflammatory scores of AIH and CHB patients; (D) Confocal staining of CD4 (in green), IL-17 (in red) and DAPI (for nuclei in blue) in the liver of AIH patients. The frequency of Th17 cells in the liver is positively correlated with hepatic inflammatory degrees (E) and fibrosis grades (F) in AIH patients.

To confirm that Th17 cells play an important role in the pathogenesis of AIH, we further investigated the distribution and frequency of Th17 cells in the liver by immunohistochemical staining of IL-17. As expected, there were very few IL-17^+^ cells in the normal livers (data not shown). There were significantly increased IL-17^+^ cells in AIH patients, compared to those with CHB (Figure 2A, B), while no difference of hepatic inflammatory degrees between AIH and CHB patients was found (Figure 2C). Most IL-17^+^ cells in AIH patients were localized in the portal tracts and lobular areas of the liver, among lymphoid infiltration. Cofocal staining showed that most IL-17^+^ cells are CD4^+^ T cells in AIH (Figure 2D), suggesting that Th17 cells are major source of IL-17 in the liver. The degree of hepatic Th17 cell infiltration was positively correlated to the degree of hepatic inflammation and the stage of hepatic fibrosis in AIH patients (Figure 2E, F). These results indicate that the infiltration of TH17 cells likely contribute to hepatic inflammation and disease progression in AIH.

**Figure 3:**
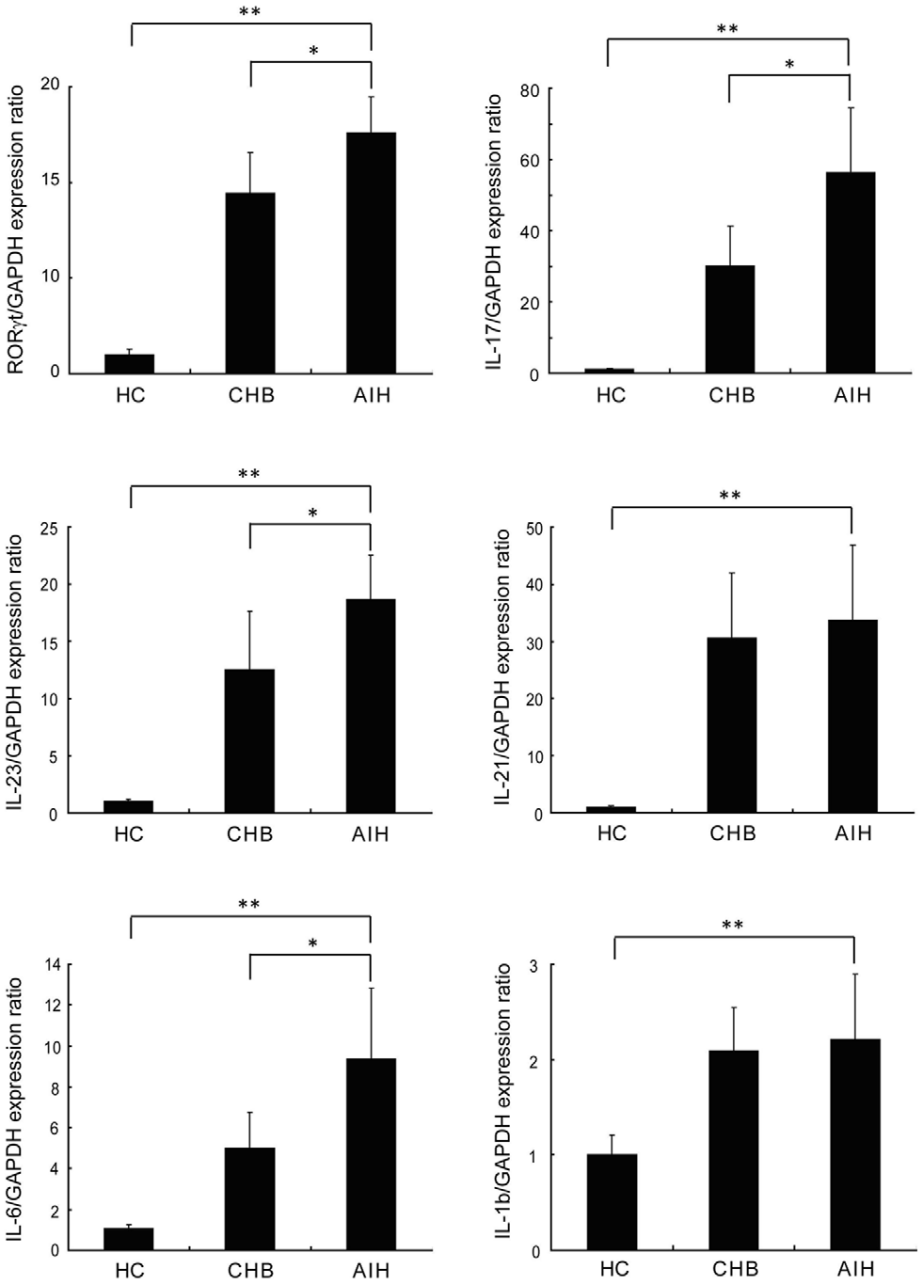
Liver tissue were obtained from healthy controls (HC, n = 5), chronic hepatitis B (CHB, n = 12) and autoimmune hepatitis (AIH, n = 28) patients. Th17 related cytokines RORγt, IL-17, IL-1β, IL-6, IL-21 and IL-23 expressions were determined by quantitative RT-PCR and normalized with GAPDH expression. *p<0.05, **p<0.01.

The function of Th17 cells relies on their ability to secret IL-17, IL-21, IL-22, IL-6 and TNFα, and their differentiation depends on specific transcription nuclear factor ROR-γt [14]. To confirm the role of Th17 cells in AIH, we evaluated Th17 cell related cytokine expressions in the liver using real-time PCR. Th17 cell related gene expressions of RORγt, IL-17, IL-23, IL-21, IL-1β, IL-6 were significantly increased in the liver of AIH patients compared to those with CHB and healthy controls (Figure 3).

### IL-17 induced IL-6 secretion in HepG2 cells through MAPK signaling pathway

**Figure 4:**
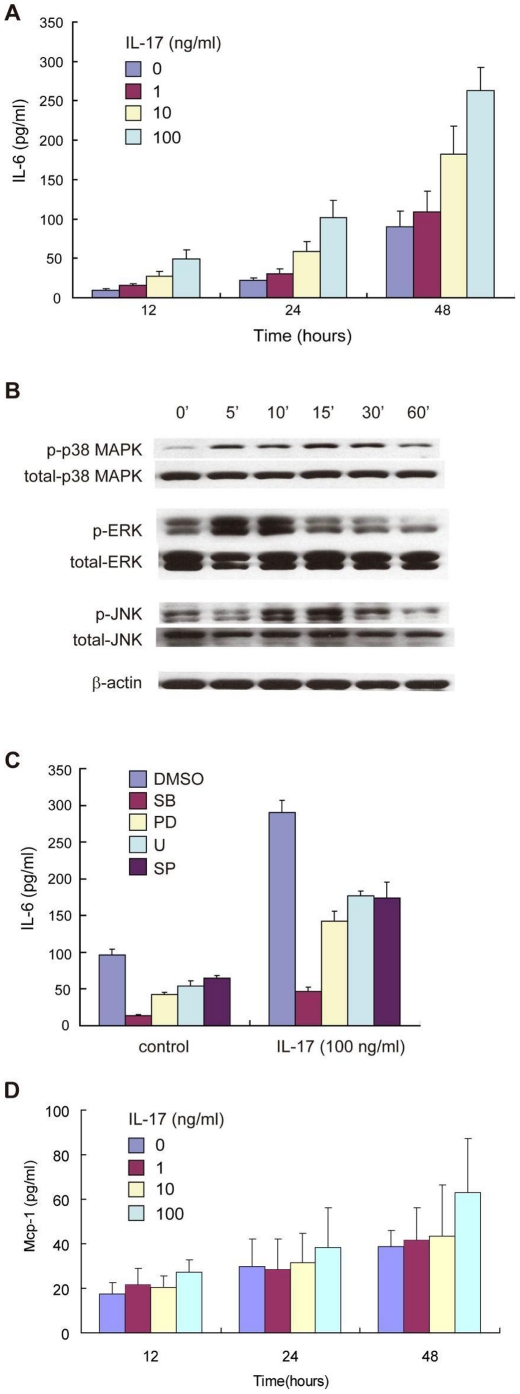
(A) IL-17 was added to HepG2 cell culture. IL-6 production by HepG2 cells in the media was measured by ELISA. (B) Total protein extract was made from HepG2 cells after stimulated with IL-17 (100 ng/ml). Western bolts were performed with antibodies specific against phosphorylated ERK1/2, p38 MAPK, JNK. The membranes were then stripped and re-probed with an antibody to total ERK, p38 MAPK and JNK. β-actin expression was determined as loading controls. (C) Specific inhibitors of MAPK signaling pathways (SB203580 for MAPK, PD98059 and U0126 for ERK, SP600125 for JNK, and DMSO as the carrier) were added to HepG2 cell culture before IL-17 stimulation. IL-6 in the media was measured by ELISA. (D) IL-17 was added to HepG2 cell culture. MCP-1 production by HepG2 cells in the media was measured by ELISA.

IL-6, in conjunction with TGF-β, promotes the generation of Th17 cells [15], while IL-17 can also stimulate IL-6 production [16]. During the liver inflammatory processes, hepatocytes are one source of IL-6 secretion in the liver in addition to monocytes and inflammatory cells [17]. We hypothesized that there is a positive-feedback loop between Th17 cells and hepatocytes through IL-6 and IL-17 interaction. To test this hypothesis, IL-17 was added to the culture medium for HepG2 cells. Although IL-17 had no effect on the growth or the differentiation of HepG2 cells, even at a high dose (data not shown), it stimulated IL-6 secretion by HepG2 cells in dose- and time-dependent manners (Figure 4A).

MAPK, ERK and JNK signaling pathways are major signaling pathways in the hepatic inflammatory process. They also regulate expression and secretion of IL-6 [18]. Previous study shows that IL-17 can activate MAPK and ERK pathway in hepatocytes [19]. To evaluate whether IL-17 stimulated IL-6 secretion in hepatocytes is MAPK pathway dependant, activation of MAPK, ERK and JNK in HepG2 cells was determined after IL-17 stimulation. IL-17 significantly enhanced the phosphorylation of MAPK, ERK and JNK in HepaG2 cells (Figure 4B), which indicate their activation. We then treated HepaG2 cells with specific inhibitors of p38 MAPK (SB203580), p42/p44 ERK (PD98059 and U0126) and JNK (SP600125) during IL-17 stimulation. All these specific inhibitors significantly attenuated IL-6 production by HepaG2 (Figure 4C). These results indicate that IL-17 induced IL-6 production in HepaG2 cells depends on activation of MAPK, ERK and JNK signaling pathways.

MCP-1 is a chemokine which activate and chemotactically attract monocytes/macrophages [20]. MCP-1 expression on hepatocytes and monocytes is increased in chronic liver diseases [21]. In our study, IL-17 did not induce MCP-1 expression of HepaG2 cells in a time- and dose-dependent manner (Figure 4D), suggesting that the effect of IL-17 on IL-6 expression of HepaG2 may be a specific process.

## Discussion

Immune reactions against host liver antigens are believed to be the major mechanism resulting in AIH. A specific autoantigenic peptide is presented to an uncommitted T helper (Th0) lymphocyte within the HLA class II molecule of an antigen-presenting cell (APC). Th0 cells, upon activation and expansion, develop into different T helper cell subsets (e.g. Th1 or Th2 cells) with different cytokine profiles and distinct effector function [3]. Th17 cells, a new T helper subset that produces IL-17, are distinct from Th1 and Th2 cells in phenotype, function, and developmental pathways [14]. Th17 cells are involved in host defense, inflammatory disease, tumor genesis, autoimmune disease, and transplant rejection through secretion of IL-17, IL-6, and other cytokines [14]. IL-17 is a pro-inflammatory cytokine that induces fibroblasts, endothelial cells, macrophages, and epithelial cells to produce other cytokines (including IL-6) and chemokines that mediate tissue infiltration and destruction. Also, IL-6, in conjunction with TGF-β, promotes the further generation of Th17 cells [15]. Th17 cells have been implemented in the pathogenesis of some liver diseases [5], but their role in AIH is less clear. In our current study, we have shown that the frequencies of Th17 cells in peripheral blood and liver of AIH patients were significantly increased compared to those of patients with other chronic inflammatory hepatitis. Furthermore, the frequency of Th17 cells is correlated with degrees of hepatic inflammation and fibrosis in AIH patients. The emerging data suggest that Th17 cells may also play an important role in liver inflammation. Liver infiltration with IL-17 secreting cells is a key feature of alcoholic hepatitis [11]. Serum Th17-related cytokines and IL-17+ lymphocytic infiltration in liver were significantly increased in patients with primary biliary cirrhosis (PBC) [22]. Most recently, Zhang J *et al* showed that Th-17 cells are increased in both peripheral blood and livers of CHB patients, and may exacerbate liver injury during chronic HBV infection [23]. Our study demonstrates that Th-17 cells are involved in both CHB and AIH. However, the frequencies of peripheral and hepatic Th17 cells are much higher in AIH patients compared to CHB patients.

In our study, we demonstrated IL-17 increases the production of IL-6 by hepatocytes, which, in turn, further stimulates Th17 cells and provides a positive feedback loop between Th17 cells and hepatocytes exacerbating the inflammatory process. It demonstrates that Th17 cell proliferation is the key trigger in the pathogenesis of AIH. In fact, it has been shown that Th17 cells play a crucial role in the pathogenesis of some autoimmune diseases, such as collagen-induced arthritis (CIA) and experimental autoimmune encephalomyelitis (EAE) [24], [25]. Accordingly, higher levels of IL-17 expression have been observed in patients with RA [26], inflammatory bowel disease (IBD) [27], and systemic lupus erythematosus (SLE) [28]. As shown in this study, neutralization of IL-17 may provide a potential therapeutic target for AIH.

Recently, it has been proposed that a numerical or functional imbalance between Th17 and regulatory T cells (Tregs) may be responsible for the development of autoimmune disease. Tregs are a group of T cells that engage in a variety of immune suppressive responses, including transplant tolerance [29], viral hepatitis [30], and autoimmune hepatitis [6]. Tregs are usually CD4^+^/CD25^+^ and express forkhead box protein 3 (Foxp3), a specific marker and the key factor in Treg development and function [31]. Intriguingly, there is a reciprocal relationship between Th17 cells and Tregs in both developmental pathways and function [9]. TGF-β can induce both anti-inflammatory Treg and pro-inflammatory Th17 depending on the local cytokine milieu. TGF-β alone can promote the expression of Foxp3 and induce the differentiation of Treg cells. The frequency of Foxp3+ cells generated in the presence of TGF-β is inversely related to the levels of IL-6, such that Foxp3+ cells were nearly extinguished in the presence of exogenous IL-6 [9]. IL-6 synergizes with TGF-β to promote the expression of RORγt, favoring Th17 differentiation. Thus, IL-6 acts as a key immunomodulator of TGF-β signaling, leading to the reciprocal differentiation of Treg and Th17. It has been demonstrated that patients with AIH have a significantly lower frequency of Treg cells at diagnosis and the frequency increases during remission on immunosuppression [6], [7], [30]. There is an inverse relationship between the percentage of Treg cells and the severity of AIH [6], [7], [30]. Our study demonstrated that IL-17 may induce hepatic IL-6 expression in AIH, which may favor proliferation of Th-17 cells while reducing the development of Tregs. Therefore, it reveals a potential mechanism that is critical in the pathogenesis of AIH by tipping the balance between Th17 and Treg cells.

In conclusion, Th17 cells and the IL-17 signaling pathway play a critical role in the pathogenesis of AIH. Restoring the imbalance among Th17 cells and Tregs by interrupting interaction between IL-17 and IL-6 may be an effective therapeutic target for autoimmune liver diseases.
